# The Presence of *Blastocystis* in Tibetan Antelope (*Pantholops hodgsonii*)

**DOI:** 10.3389/fcimb.2021.747952

**Published:** 2021-09-29

**Authors:** Hong-Li Geng, Yu-Zhe Sun, Jing Jiang, He-Ting Sun, Yuan-Guo Li, Si-Yuan Qin, Zhen-Jun Wang, Tao Ma, Jun-Hui Zhu, Nian-Yu Xue, Hong-Bo Ni

**Affiliations:** ^1^ College of Veterinary Medicine, Qingdao Agricultural University, Qingdao, China; ^2^ College of Life Sciences, Changchun Sci-Tech University, Shuangyang, China; ^3^ General Monitoring Station for Wildlife-Borne Infectious Diseases, State Forestry and Grass Administration, Shenyang, China; ^4^ Key Laboratory of Zoonosis Research, Ministry of Education, College of Veterinary Medicine, Jilin University, Changchun, China; ^5^ College of Animal Science and Veterinary Medicine, Heilongjiang Bayi Agricultural University, Daqing, China

**Keywords:** *Blastocystis*, prevalence, subtypes, Tibetan antelope (*Pantholops hodgsonii*), PCR

## Abstract

*Blastocystis* is a protozoan that parasitizes the intestines. A number of hosts of *Blastocystis* have been found, including human and animals. However, there has been no research on the prevalence of *Blastocystis* in Tibetan antelope. Here, a molecular test was performed using 627 Tibetan antelope fecal samples collected on Tibet in China from 2019 to 2020. The result showed that 30 (4.8%) samples were *Blastocystis* positive. The highest prevalence of *Blastocystis* was in Shuanghu County (25/209, 12.0%), followed by Shenza County (2/103, 1.9%), Nyima County (3/182, 1.6%), and Baigoin County (0/133, 0.0%). In addition, logistic regression analysis showed that the gender, sampling year, and area of Tibetan antelope were risk factors for *Blastocystis* prevalence. Three subtypes (ST10, ST13, and ST14) of *Blastocystis* were found in Tibetan antelope through a subtype sequence analysis, and ST13 was identified to be the dominant subtype. This is the first investigation for the infection of *Blastocystis* in Tibetan antelope. Collectively, the data in this study have expanded the host range of *Blastocystis* and provided basic information for the distribution of *Blastocystis* subtypes, which could support the prevention of *Blastocystis* infection in wild animals.

## Introduction

*Blastocystis* is a protozoan that parasitizes the intestines (Jiménez et al., 2019; Paik et al., 2019). It can infect a variety of hosts, such as mammals, amphibians, birds, and insects (Zhu et al., 2020). *Blastocystis* is transmitted through the fecal–oral route or water and food between susceptible hosts (Asghari et al., 2019; Deng et al., 2019). Hosts infected with *Blastocystis* could develop clinical signs, e.g., diarrhea, abdominal pain, and vomiting. Immunocompromised individuals are more susceptible to *Blastocystis* (Wang et al., 2018a; Paik et al., 2019).

*Blastocystis* was first isolated from animal feces in 1911 (Alexeieff, 1911). Since then, more and more animals and humans, such as cattle, deer, sheep, and goats, were identified to be the hosts of *Blastocystis* (**Table 1**). In China, *Blastocystis* was first reported in children in 1990 (Li et al., 1990). A large number of investigations regarding *Blastocystis* prevalence in different hosts were performed previously (Song et al., 2017; Zhao et al., 2017; Wang et al., 2018a; Wang et al., 2018b). So far, the infection of *Blastocystis* has been reported in many animals, including domestic and wild animals (Zhao et al., 2017; Wang et al., 2018a).

**Table 1:** Subtypes and prevalence of *Blastocystis* sp. detected from the ruminants worldwide (2010–2021).

Host	No. tested	No. positive	Prevalence (%)	Location	Subtypes (STs) identified	Reference
Sheep	832	50	6.00%	China	ST5, ST10, ST14	Li et al., 2018
Sheep	109	6	5.50%	China	ST1, ST5, ST10, ST14	Wang et al., 2018a
Sheep	100	32	32.00%	Iran	ST3, ST5, ST7, ST14	Salehi et al., 2021
Goat	781	2	0.30%	China	ST1	Li et al., 2018
Goat	236	73	30.90%	Malaysia	ST1, ST3, ST6, ST7	Tan et al., 2013
Goat	400	3	0.75%	Nepal	NA	Ghimire and Bhattarai, 2019
Goat	38	36	94.70%	Thailand	ST10, ST12, ST14	Udonsom et al., 2018
Goat	789	458	58.00%	China	ST1, ST3, ST4, ST5	Song et al., 2017
Cattle	147	14	9.52%	China	ST3, ST10, ST14	Wang et al., 2018a
Cattle	22	5	22.70%	UAE	ST10	AbuOdeh et al., 2019
Cattle	28	6	21.40%	Brazil	NA	Moura et al., 2018
Cattle	42	21	50.00%	Thailand	ST10, ST12	Udonsom et al., 2018
Cattle	80	9	11.30%	Turkey	ST10, ST14	Aynur et al., 2019
Cattle	526	54	10.30%	China	ST4, ST5, ST10, ST14	Zhu et al., 2017
Cattle	47	9	19.20%	USA	ST10, ST14	Fayer et al., 2012
Cattle	196	19	9.60%	Iran	ST3, ST5, ST6	Badparva et al., 2015
Cattle	31	7	22.60%	England	ST1, ST5, ST10	Alfellani et al., 2013
Cattle	25	20	80.00%	Colombia	ST1, ST3	Ramírez et al., 2014
Cattle	36	15	41.70%	Libya	ST5, ST10, ST14	Alfellani et al., 2013
Cattle	75	25	33.30%	Iran	ST5, ST10	Sharifi et al., 2020
Cattle	29	10	34.50%	Malaysia	NA	Hemalatha et al., 2014
Cattle	40	14	35.00%	Iran	ST10, ST14	Rostami et al., 2020
Cattle	1512	101	6.70%	Korea	ST1,5,10,14	Lee et al., 2018
Cattle	133	72	54.10%	Japan	ST14	Masuda et al., 2018
Cattle	254	161	63.40%	Lebanon	ST1, ST3, ST5, ST10, ST14	Greige et al., 2019
Cattle	110	6	5.40%	Malaysia	NA	Abd et al., 2019
Cattle	1027	278	27.10%	China	ST10	Ren et al., 2019
Cattle	500	47	9.40%	Indonesia	NA	Hastutiek et al., 2019
Cattle	2539	73	2.90%	USA	ST3, ST4, ST5, ST10, ST14	Maloney et al., 2019
Cattle	120	30	25.00%	Malaysia	ST1, ST3, ST4, ST5, ST10, ST14	Kamaruddin et al., 2020
Cattle	108	108	100.00%	Indonesia	ST10	Suwanti et al., 2020
Reindeer	104	7	6.70%	China	ST10, ST13	Wang et al., 2018b
Sika deer	82	12	14.60%	China	ST10, ST14	Wang et al., 2018b
Water deer	125	51	40.80%	Korean	ST4, ST14	Kim et al., 2020
Red deer	48	0	0.00%	China	NA	Wang et al., 2018b
Spotted deer	30	1	3.30%	Bangladesh	ST14	Li et al., 2019
Sika deer	132	60	45.50%	Japan	ST14	Shirozu et al., 2021
White tailed-deer	80	71	88.80%	USA	ST1, ST3, ST4, ST10, ST14, ST21, ST23, ST24, ST25, ST26	Maloney and Santin, 2021

UAE, United Arab Emirates; NA, not available.

To date, approximately 29 proposed *Blastocystis* subtypes have been identified in a large number of literatures (Ma et al., 2020). ST1-9 and ST12 subtypes were identified in humans, while ST10-17 and ST21-28 subtypes were detected in animals (Stensvold and Clark, 2016; Ning et al., 2020; Hublin et al., 2021). Of note, some subtypes were identified in both humans and animals, such as ST1, ST3, and ST5 subtypes (Song et al., 2017; Wang et al., 2018a). ST4 was found in deer (Wang et al., 2018b; Kim et al., 2020; Shirozu et al., 2021), and ST12 was found in yaks in the plateau area (Ren et al., 2019).

Tibetan antelope (*Pantholops hodgsonii*) belongs to genus Pantholops, family Bovidae, order Cetartiodactyla according to the IUCN Red List in 2016 (IUCN SSC Antelope Specialist Group, 2016). Tibetan antelope is one of the most rare and endangered wild animals. There are approximately 100,000 to 150,000 Tibetan antelope in India and China (IUCN SSC Antelope Specialist Group 2016). Tibetan antelope can carry various pathogens, such as *Mycoplasma capricolum* subspecies, *capripneumoniae* (Mccp) (Yu et al., 2012), and *Escherichia coli* (Bai et al., 2016).

However, the existing data indicate that sheep may carry several potential *Blastocystis* subtypes, including ST1, ST3, ST4, ST5, ST6, and ST7 (Tan et al., 2013; Song et al., 2017; Wang et al., 2018a; Salehi et al., 2021). So far, studies on the prevalence and subtype diversity of *Blastocystis* in Tibetan antelope are unknown and the relevant public health impact is still unclear. This study provides important information on the diversity of *Blastocystis* subtypes in Tibetan antelope, and would help determine the role of Tibetan antelope in the transmission of *Blastocystis* to humans and other animals.

## Materials and Methods

### Specimen Collection

**Figure 1:**
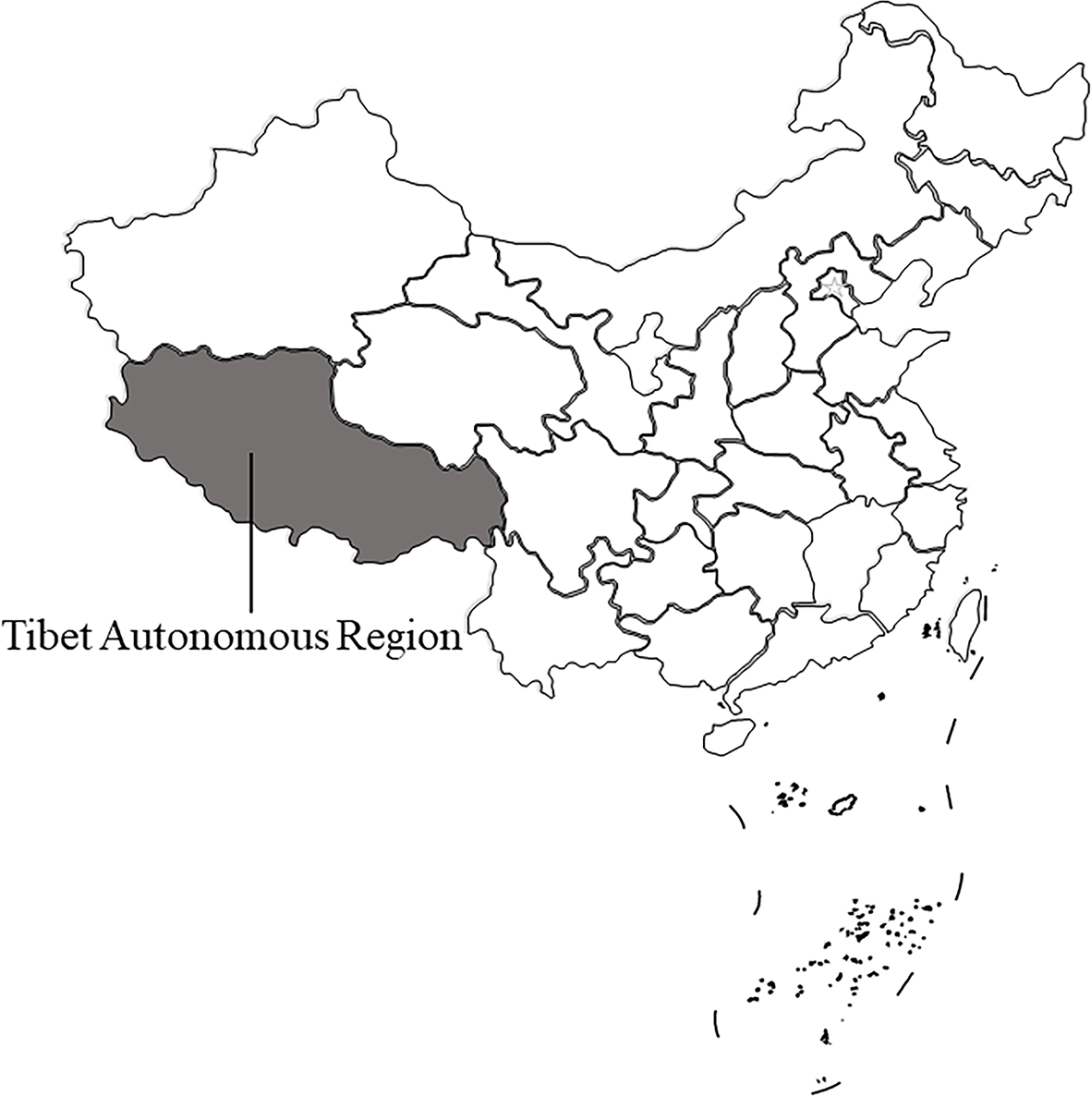
A map of Tibet Autonomous Region, China, in which Tibetan antelope are sampled.

From August 2019 to September 2020, the feces of 627 wild Tibetan antelope was collected in four areas in Tibet (**Table 2** and **Figure 1**). This study randomly observed Tibetan antelope in the field. Fresh fecal samples were put into a PE glove immediately after defecation onto the ground, and then were placed into ice boxes and transported to the laboratory. This study was approved by the Ethics Committee of Jilin University. Appropriate permission was obtained from the General Monitoring Station for Wildlife-Borne Infectious Diseases, State Forestry and Grass Administration.

**Table 2:** Factors in the sampling site of different seasons in tibet.

Season	Longitude	Latitude	Altitude	Temperature	Humidity	Climate
Summer (August)	31°29′	92°04′	4,507 m	9.0°C	68 mm	Plateau alpine climate
Autumn (September)				6.1°C	70 mm	

### DNA Extraction and PCR Amplification

Genomic DNA was extracted using the E.Z.N.A.^®^ Stool DNA Kit (Omega Biotek Inc., Norcross, GA, USA) according to the manufacturer’s instructions and stored at −20°C until PCR amplification. SSU rRNA gene was the target for PCR analysis using primers RD5 (5′-ATCTGGTTGATCCTGCCAGT-3′) and BhRDr (5′-GAGCTTTTTAACTGCAACAACG-3′) as described previously to amplify an approximately 600-bp region (Scicluna et al., 2006). Positive and negative controls were included in each test. PCR products were observed using UV light after electrophoresis at a 1.5% agarose gel containing ethidium bromide.

### Sequence and Phylogenetic Analyses

The *Blastocystis*-positive PCR products were sent to Sangon Biotech Company (Shanghai, China) for sequencing. The sequence accuracy was confirmed by bidirectional sequencing. The sequencing was re-performed if variation was present in the previous result. Basic Local Alignment Search Tool (BLAST) (http://www.ncbi.nlm.nih.gov/blast/) was employed to compare consensus sequences with the similar sequences on GenBank. The subtypes of *Blastocyst* isolates were determined through the online platform PubMLST (https://pubmlst.org/bigsdb?db=pubmlst_blastocystis_seqdef). The obtained sequences were aligned using ClustalX 1.83 program. The alignment was trimmed using the trimAI v1.2 software (http://trimal.cgenomics.org/downloads) (Li et al., 2019). All positions with gaps were eliminated, and 104 unambiguously aligned sites were used for phylogenetic inference. The maximum likelihood (ML) method (Kimura two-parameter model) was employed to reconstruct phylogenetic trees by using MEGA X. Representative nucleotide sequences were submitted to GenBank under accession numbers: MZ444657–MZ444662.

### Statistical Analysis

The variation of *Blastocystis* prevalence (*y*) in Tibetan antelope on the basis of sampling year (*x*1), gender (*x*2), and collecting region (*x*3) was analyzed with *χ* ^2^ test using SAS version 9.4 (SAS Institute Inc., USA). In the multivariable regression analysis, each of the variables was independently contained in the binary Logit model. The best model was judged by Fisher’s scoring algorithm. All tests were two-sided, and the results were considered statistically significant when *p* < 0.05. To explore the association between *Blastocystis* prevalence and the investigated factors, the odds ratios (ORs) and their 95% confidence intervals (95% CIs) were calculated.

## Results

### Prevalence of *Blastocystis* sp.

**Figure 2:**
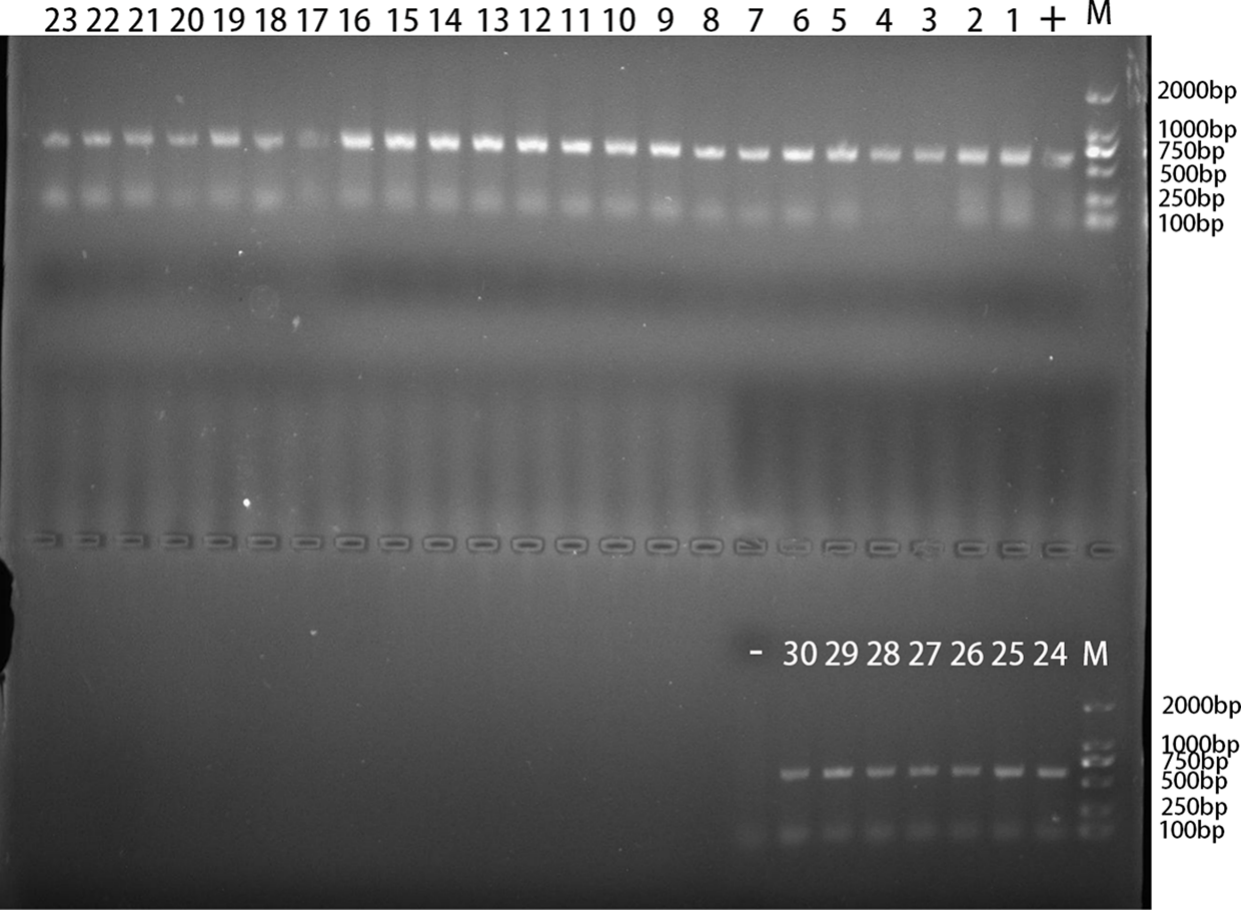
The PCR amplification result of *Blastocystis* rRNA gene. “1–30”: 30 positive samples of *Blastocystis* in TA25, TA26, TA72, TA100, TA103, TA105, TA107, TA108, TA111, TA118, TA124, TA128, TA130, TA131, TA147, TA148, TA156, TA157, TA169, TA175, TA214, TA216, TA228, TA230, TA232, TA235, TA238, TA252, TA291, and TA307; “-”: negative control; “+”: positive control; “M”: DL2000 Marker.

In the present study, 30 out of 627 Tibetan antelope feces were identified to be *Blastocystis* positive (**Figure 2**). The infection rate of *Blastocystis* in Tibetan antelope in 2019 (0.6%, 2/322) was lower than that (9.2%, 28/305) in 2020. The prevalence of *Blastocystis* in different investigated counties ranged from 0% to 12% (**Table 3**). *Blastocystis* were detected in all three counties, except for Baigoin County. The highest prevalence of *Blastocystis* was in Shuanghu County (25/209, 12.0%), followed by Shenza County (2/103, 1.9%) and Nyima County (3/182, 1.6%; **Table 3**). The infection rate of *Blastocystis* in Tibetan antelope in females (2.7%, 8/300) was significantly lower than that (6.7%, 22/327) in males (*p* = 0.017).

**Table 3:** Occurrence and subtype distribution of *Blastocystis* sp. in tibetan antelope (*Pantholops hodgsonii*).

Factor	Category	No. tested	No. positive	Prevalence (%) (95% CI)	*p*-value	OR (95% CI)	Subtypes (no.)
Gender	Female	300	8	2.7 (0.8–4.5)	0.017	Reference	ST10 (1); ST13 (6); ST14 (1)
	Male	327	22	6.7 (4.0–9.4)		2.63 (1.15–6.00)	ST10 (3); ST13 (18); ST14 (1)
Sampling year	2019	322	2	0.6 (0.0–1.5)	<0.01	Reference	ST13 (2)
	2020	305	28	9.2 (5.9–12.4)		16.17 (3.82–68.50)	ST10 (4); ST13 (22); ST14 (2)
Region	Nyima County	182	3	1.6 (0.0–3.5)	<0.01	Reference	ST13 (3)
	Shuanghu County	209	25	12.0 (7.6–16.4)		8.11 (2.41–27.33)	ST10 (3); ST13 (20); ST14 (2)
	Shenza County	103	2	1.9 (0.0–4.6)		1.18 (0.19–7.19)	ST10 (1); ST13(1)
	Baingoin County	133	0	0.0 (0.0–0.0)		–	–
Total		627	30	4.8 (3.1–6.5)			ST10 (4); ST13 (24); ST14 (2)

### Risk Factors of *Blastocystis* sp.

To expose gender, sampling year, and collecting region of Tibetan antelope, and *Blastocystis* prevalence, univariate analysis was also conducted in the present study (**Table 3**). A Fisher’s scoring method-based positive stepwise logistic regression analysis was performed to estimate the influence of multiple variables on *Blastocystis* infection. Only one variable was found to have effects on the *Blastocystis* infection in the final model, as described by the equation: *y* = 1.3138*x*3 + 0.7097. Collecting region had a positive impact on the risk of *Blastocystis* infection with the OR of 3.720 (95% CI 2.061–6.715). Nyima County (1.6%, 95% CI 0.0–3.5) was considered to have lower prevalence than Shuanghu County (OR = 8.11, 95% CI 2.41–27.33) and Shenza County (OR = 1.18, 95% CI 0.19–7.19) (**Table 3**).

### Distribution and Phylogenetic Analysis of *Blastocystis* Subtypes

Three *Blastocystis* subtypes (ST10, ST13, and ST14) were detected in this study. Among them, the ST13 subtype was found in 24 individuals and was widely distributed in different gender subgroups, sampling years, and collecting regions. In the sampling years, all three *Blastocystis* subtypes appeared in Tibetan antelope in 2020, and only ST13 was found in the Tibetan antelope in 2019 (**Table 3**). In the gender subgroups, although all of the three subtypes appeared in the Tibetan antelope, the infection of ST10 and ST13 in males was higher than that in females. Tibetan antelope in Shuanghu County was found to be infected with three *Blastocystis* subtypes in the regional subgroups. However, only the ST13 subtype was found in the Tibetan antelope in Nyima County (**Table 3**).

**Figure 3:**
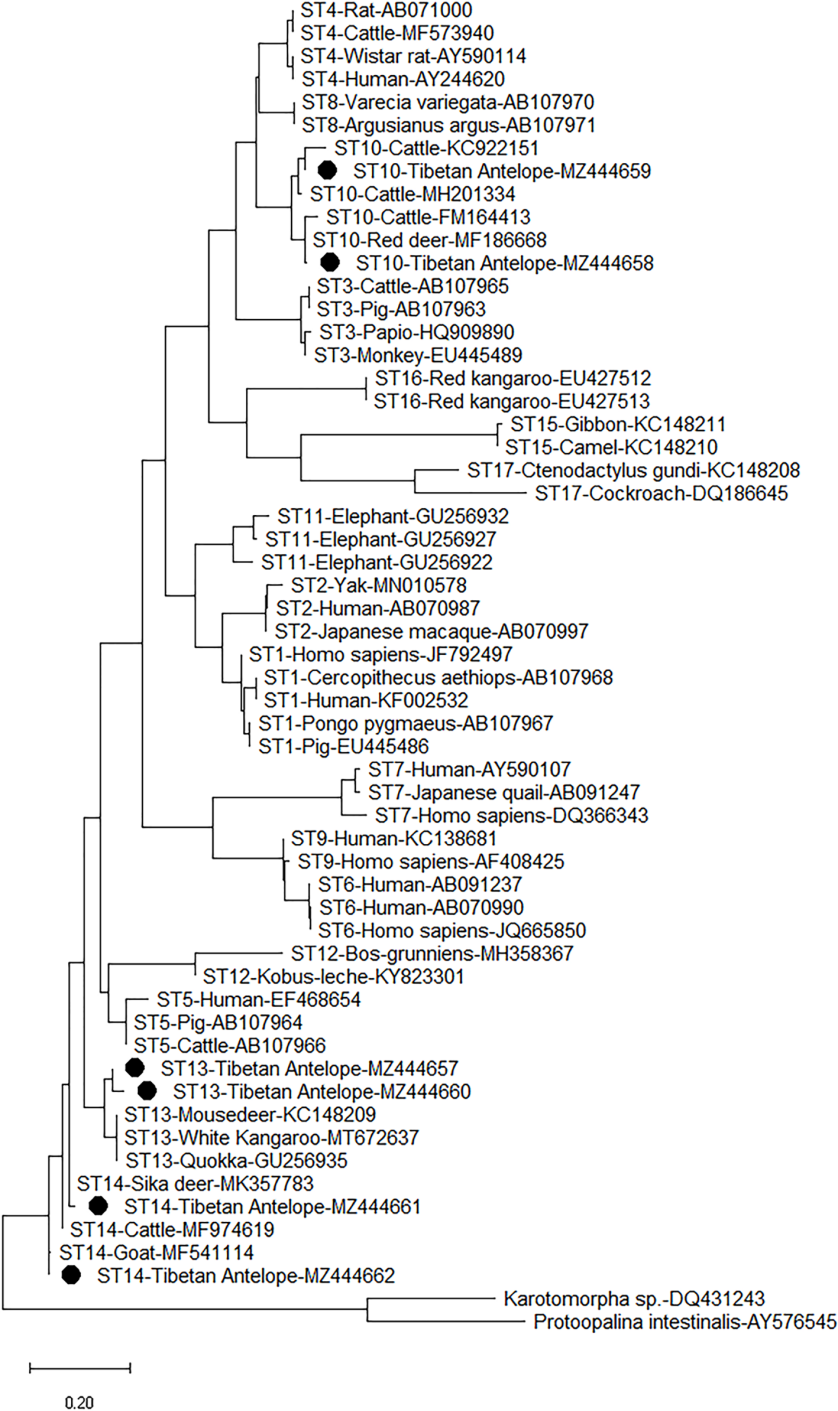
Phylogenetic analyses of *Blastocystis* using (ML) method (Kimura two-parameter model). Bootstrap values below 50% from 1,000 replicates are not shown. *Blastocystis* isolates identified in the present study are indicated by solid circles.

The six representative sequences in this study and 49 sequences on GenBank were used to construct a phylogenetic tree. According to the phylogenetic tree analysis, the sequences of the three subtypes (ST10, ST13, and ST14) obtained from this study were clustered with their reference subtypes (**Figure 3**). The sequence of ST10 isolate has 99% homology with that of ST10 isolated from sika deer (MK930358) and sheep (MW850529). The sequence identified as ST13 in this study has a high degree of homology (99%) with the sequence identified in white Kangaroo (MT672637) and reindeer (MH325366). The ST14 sequence has 98% homology with the known reference sequence identified in sheep (MF186707).

## Discussion

The overall infection rate of *Blastocystis* in Tibetan antelope was 4.8% (30/627) in Tibet, which was lower than the prevalence of 5.5% (6/109) and 6.0% (50/832) identified in sheep (Wang et al., 2018a; Li et al., 2018) in China and 19.3% (9/150) and 32.0% (32/100) in sheep in Iran (Rostami et al., 2020; Salehi et al., 2021). The difference in the prevalence of *Blastocystis* may be related to the living environment and geographical factors of different countries (Tan, 2008). The infection rate of *Blastocystis* in different species is different. For example, the prevalence of Tibetan antelope in this study and sheep, goats, and cattle in other studies were 4.8% (30/627), 0.75% (3/400) (Ghimire and Bhattarai, 2019), and 14.43% (72/500) (Hastutiek et al., 2019), respectively. The results showed that the prevalence of *Blastocystis* might be related to the sensitivity of animals to *Blastocystis*. Therefore, future research should collect more samples to better understand the population characteristics of *Blastocystis* in Tibetan antelope.

The average temperature in Baingoin County is −17.1°C annually, which is much lower than that of other counties. The survival of *Blastocystis* may be affected by the low-temperature environment in Baingoin County. *Blastocystis* might survive in warm and humid environment (Sari et al., 2021). This is probably the reason why the infection rate of *Blastocystis* in Baingoin County (0.0%, 0/133) was significantly lower than that of the other three counties.

Previous studies have shown that the infection rate of *Blastocystis* in males (4.8%, 25/517) was higher than that in females (3.1%, 9/291) in cattle (Lee et al., 2018) and in sambar (males: 38.2%, 21/55 *vs.* females: 23.3%, 7/30) (Kim et al., 2020). This study also found that the infection rate of *Blastocystis* (6.7%, 22/327) was higher in males than in females (2.7%, 8/300) of Tibetan antelope. This may be due to the fact that males have a wider range of activities than females, and have a relatively higher chance for contacting with cysts.

At present, 29 proposed *Blastocystis* subtypes have been identified (Maloney and Santin, 2021). Among them, ST1, ST3, ST5, ST10, and ST14 subtypes were detected in sheep and goats (Song et al., 2017; Li et al., 2018; Wang et al., 2018a), among which ST10 and ST14 were the most common subtypes (Fayer et al., 2012; Zhao et al., 2017; Hublin et al., 2021). ST10 and ST14 were also detected in Tibetan antelope. However, it is worth noting that ST10 (*n* = 4) and ST14 (*n* = 2) were not common in the samples of Tibetan antelope in this study. On the contrary, ST13 (*n* = 24) represented the infection trend of *Blastocystis* in Tibetan antelope. ST13 subtype is a relatively rare subtype. ST13 has been detected in deer, flying squirrels, kangaroo, monkeys, and other animals (Parkar et al., 2010; Alfellani et al., 2013; Wang et al., 2018b; Li et al., 2019; Xiao et al., 2019). Compared with domestic animals, ST13 may be more common in wild animals. Therefore, the follow-up research should focus on the distribution of *Blastocystis* genotypes in wild animals.

In summary, this is the first report of *Blastocystis* infection in Tibetan antelope in Tibet, China. The total prevalence of *Blastocystis* was 4.11% (30/627). Moreover, ST10, ST13, and ST14 subtypes were found in Tibetan antelope, among which ST13 was the dominant subtype. These results not only expanded the knowledge of hosts of *Blastocystis*, but also provided data for further studies on the distribution of *Blastocystis* subtypes in Tibetan antelope, and also provided data supporting for the prevention of *Blastocystis* infection in wild animals.

## Data Availability Statement

The datasets presented in this study can be found in online repositories. The names of the repository/repositories and accession number(s) can be found in the article/supplementary material.

## Ethics Statement

The animal study was reviewed and approved by the Ethics Committee of Jilin University.

## Author Contributions

H-BN and JJ conceived and designed the study and critically revised the manuscript. S-YQ, H-TS, J-HZ, Z-JW, and TM collected the samples. H-LG, Y-ZS, Y-GL, and N-YX performed the experiments. H-LG and Y-ZS analyzed the data and drafted the manuscript. All authors contributed to the article and approved the submitted version.

## Funding

This work was supported by the Research Foundation for Distinguished Scholars of Qingdao Agricultural University (665-1120046).

## Conflict of Interest

The authors declare that the research was conducted in the absence of any commercial or financial relationships that could be construed as a potential conflict of interest.

## Publisher’s Note

All claims expressed in this article are solely those of the authors and do not necessarily represent those of their affiliated organizations, or those of the publisher, the editors and the reviewers. Any product that may be evaluated in this article, or claim that may be made by its manufacturer, is not guaranteed or endorsed by the publisher.
